# Night and shift work characteristics and incident ischemic heart disease and atrial fibrillation among healthcare employees – a prospective cohort study

**DOI:** 10.5271/sjweh.4045

**Published:** 2022-10-01

**Authors:** Manzur Kader, Jenny Selander, Tomas Andersson, Maria Albin, Theo Bodin, Mikko Härmä, Petter Ljungman, Carolina Bigert

**Affiliations:** 1 Institute of Environmental Medicine, Karolinska Institutet, Stockholm, Sweden; 2 Centre for Occupational and Environmental Medicine, Region Stockholm, Stockholm, Sweden; 3 Finnish Institute of Occupational Health, Helsinki, Finland; 4 Department of Cardiology, Danderyd University Hospital, Sweden

**Keywords:** arrhythmia, cardiovascular disease, coronary heart disease, night work, occupational exposure, occupational health, quick returns, register data, Stockholm, Sweden, work schedule

## Abstract

**Objective:**

This study aimed to examine the effects of various aspects of night and shift work on the risk of incident ischemic heart disease (IHD) and atrial fibrillation (AF) using detailed and registry-based exposure data.

**Methods:**

This prospective cohort study included >30 300 healthcare employees (eg, nurses, nursing assistants) employed for at least one year in Region Stockholm 2008–2016. Information on daily working hours was obtained from a computerized administrative employee register and outcomes from national and regional registers. Using discrete-time proportional hazard models, we analyzed the outcomes as functions of working hour characteristics the preceding year, adjusted for sex, age, country of birth, education, and profession.

**Results:**

We observed 223 cases of IHD and 281 cases of AF during follow-up 2009–2016. The risk of IHD was increased among employees who the preceding year had permanent night shifts compared to those with permanent day work [hazard ratio (HR) 1.61, 95% confidence interval (CI) 1.06–2.43] and among employees working night shifts >120 times per year compared to those who never worked night (HR 1.53, 95% CI 1.05–2.21). When restricted to non-night workers, the risk of IHD was increased for employees having frequent quick returns from afternoon shifts. No increased risks were observed for AF.

**Conclusions:**

Night work, especially working permanent night shifts and frequent night shifts, is associated with an increased risk of incident IHD but not AF. Moreover, frequent quick returns from afternoon shifts (among non-night workers) increased IHD risk. Organizing work schedules to minimize these exposures may reduce IHD risk.

Healthcare employees have shift work schedules involving irregular or unusual hours of work to provide services around-the-clock. Recent European data showed that about 40% of healthcare employees worked on shifts, such as permanent shifts of mornings, afternoons, or nights, alternating or rotating shifts, and 14% reported usual weekly working hours longer than the standard 40-hour workweek. Moreover, 23% of employees in all sectors combined had a short recovery time (<11 hours) between two shifts (1).

Cardiovascular diseases (CVD) and especially ischemic heart disease (IHD) are leading causes of morbidity and mortality worldwide (2). Atrial fibrillation (AF) is the world’s most common heart rhythm disorder, associated with increased morbidity and mortality. AF is one of the leading risk factors for ischemic stroke and is also associated with developing heart failure and dementia (3, 4). Both IHD and AF share many of the same risk factors, such as increased age, obesity, hypertension, and physical inactivity (5). Shift work has been hypothesized to increase the risk of chronic diseases like CVD through several pathways including circadian rhythm disruption, which may affect glucose and lipid metabolism, appetite regulation, inflammation, hormone secretion, hypertension, atherosclerosis, and autonomic nervous system imbalance (6–8). In addition, shift workers may have poorer psychosocial working conditions, such as job strain and social stress (6, 9), and poorer lifestyle behaviors, such as poor diet, physical inactivity, and smoking habits compared to non-shift workers (9–11), which can, in turn, increase the risk of CVD.

Previous studies of the association between shift work, long working hours, and the risk of CVD mostly focused on IHD and have been summarized in several systematic reviews and meta-analyses (12–17). The first systematic review of 16 epidemiological studies by Frost et al (12) found limited epidemiological evidence for a causal relationship between shift work, mainly evening, night, and rotating shift work, and the risk of IHD. In a recent meta-analysis of 31 independent results, Cheng et al (17) found a pooled risk ratio of 1.13 [95% confidence interval (CI) 1.08–1.20] for IHD. However, data on the association between night and shift work and AF are very limited. AF episodes follow a circadian rhythm and occur most commonly in the early morning and the early evening hours (18). A recent cohort study of the UK Biobank population shows that self-reported night shift work of >10 years and working an average of 3–8 nights per month increased the risk of AF (19). Kivimäki et al (20), in a prospective multi-cohort study reported that those working ≥55 hours per week had an approximately 40% higher risk of AF than those working a standard 35–40-hour work week. However, in both studies, working hour information was self-reported and assessed at the baseline only, which may lead to both differential and non-differential misclassification of the exposure. Relying on self-reported information on working hours or lack of longitudinal exposure information is also a major limitation of several of the aforementioned studies on shift work and CVD. Further cohort studies of shift workers have been recommended to use detailed and more precise exposure information on working hours and hereby overcome the limitations related to exposure assessment in prior studies (21–23). Therefore, in the present study, we used registry-based exposure data from a computerized HR administrative system, including detailed individual information on working hours day-by-day for all participants in a cohort of healthcare employees in Stockholm. This study aimed to examine the effects of various aspects of night and shift work on the risk of incident IHD and AF in a prospective design.

## Methods

### Study design and participants

**Figure 1:**
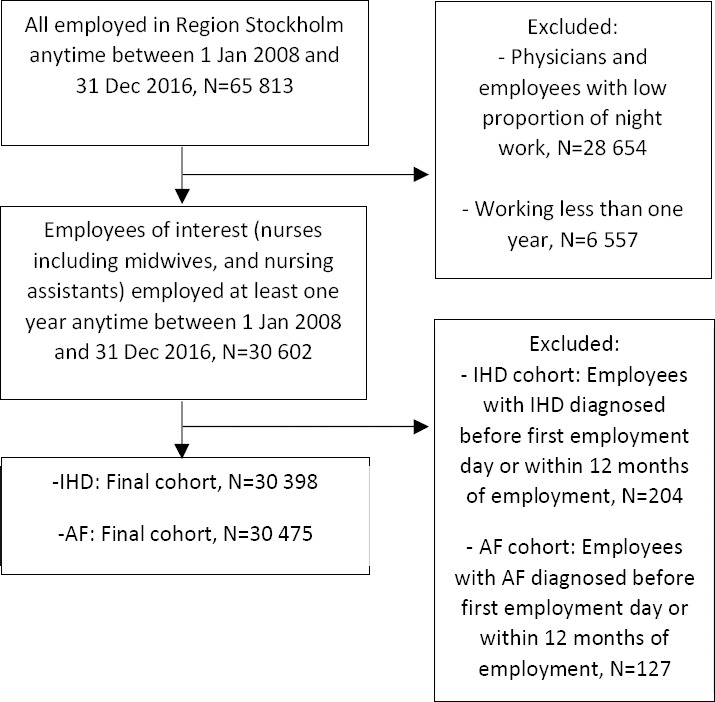
Flowchart for the inclusion and exclusion procedure, cohort for ischemic heart disease (IHD) and cohort for Atrial fibrillation (AF).

This study is a prospective cohort study of healthcare professionals who were employed in in- or outpatient care services for at least one year anytime between 1 January 2008 and 31 December 2016, identified from a computerized administrative employee register (HEROMA) in Region Stockholm, Sweden. We restricted the study to employees in Region Stockholm who often work night shifts, which includes nurses, including midwives, nursing assistants, or related professions (eg, accommodation assistants, caregivers, personal assistants) (N=30 602). Physicians were not included in the present analysis due to less detailed information on working hours and night work. From the total 30 602 participants, we created an analytical subsample investigating the risk of IHD by excluding 204 employees with a previous diagnosis of IHD before the first employment day (based on data back to 1998) or within the first 12 months of employment. Correspondingly in the AF subsample, we excluded 127 employees with a previous AF diagnosis before the first employment day or within the first 12 months of employment. Thus, the final samples comprised 30 398 participants (26 624 women and 3774 men) in the IHD cohort and 30 475 participants (26 680 women and 3795 men) in the AF cohort. A flowchart for the inclusion and exclusion procedure for both IHD and AF cohorts is presented in figure 1.

### Assessment of exposure

HEROMA in Region Stockholm provided information on working hours. We collected detailed individual information on working hours day-by-day for all employed between 1 January 2008 and 31 December 2016, including information on the occupation for each working period. For each working shift, there is information on the exact start- and end times. We excluded work shifts with a duration of <4 hours (0.7%). The method for aggregation and characterization of working time patterns based on register-based working hours for healthcare employees was previously evaluated in Finland (24), and was described specifically for HEROMA in our two studies investigating shift and night work and the risk of cerebrovascular disease (25) and preterm birth (26).

### Classification of exposure

Detailed information on the exposure classification has been described previously (25). Briefly, based on all work schedules, three types of shifts were identified: day shifts (starts after 06:00 and ends no later than 18:00); afternoon shifts (starts after 12:00 and ends later than 18:00, but not a night shift); and night shifts (≥3 hours of work within 22:00–06:00). Furthermore, the cohort participants were, for each year, classified as (i) permanent daytime workers, (ii) persons working daytime and afternoon shifts but not night shifts, (iii) persons working various shift types including night shifts, and (iv) persons working permanent night shifts. The exposure classification was assessed annually to consider variations over time.

For each year, the cut-off points in classifying exposures were chosen based on 1–25^th^, 25^th^–50^th^, 50^th^–75^th^ and >75^th^ percentile in the distribution of the exposed group. Thus, we calculated the frequency of night shifts, quick returns from night shifts (<28 hours between the end of a night shift and the beginning of the following shift), and quick returns from afternoon shifts (<11 hours between the end of an afternoon shift and the beginning of the following shift) per year. Regarding long working hours, we calculated the frequency of long (>45 hours) working weeks per year. We also conducted analyses based on continuous measures (test for trend).

### Assessment of the outcome

Incident IHD and AF events were defined as the first occurrence of events as described by the International Classification of Diseases, tenth revisions (ICD-10); IHD (ICD: I20-25), and AF or atrial flutter (ICD: I48). The national patient register at the National Board of Health and Welfare (inpatient and specialized outpatient care) and the regional database from general practice in Stockholm (VAL database) provided data on IHD and AF. The VAL database records the diagnoses of non-hospitalized patients from both inpatient care and outpatient contacts within the Region Stockholm. We included the IHD or AF diagnosis that appeared first in either of the two registers, during the follow-up period between 1 January 2009 and 31 December 2016. The employees were at risk from one year after the start of employment until diagnosis, death, or end-of-follow-up, whichever came first.

### Covariates

Information on age, sex, education, and country of birth was obtained from the register of the total population at Statistics Sweden.

### Data analysis

To examine the effect of different types of shift characteristics on the risk of IHD and AF, we used discrete-time proportional hazard models, stratifying the person-time experience of the cohort by follow-up year, starting the assessment of risk one year after the first employment day (27). In the estimations of hazard ratios (HR), we adjusted for several factors that might affect the risk, such as age (continuous), sex, education (higher education (university ≥3 years), upper secondary or elementary school or less), country of birth (Sweden, Nordic countries except Sweden, Europe except Nordic countries, other countries) and profession (nurses including midwives, nursing assistants or other related professions). Moreover, country of birth was adjusted for as a proxy measure for ethnicity, and educational level and profession were adjusted for as a proxy for socioeconomic status. Age and exposure variables were treated as time-dependent variables updated for each year of follow-up, whereas sex, education, country of birth, and profession remained the same during all the follow-up years. Additionally, some analyses were also adjusted for the number of total night shifts per year in order to disentangle the effect of intensity of night work in general from the effect of working consecutive night shifts or having quick returns from night shifts.

We estimated HR with 95% CI in the association between different shift work characteristics and incident IHD and AF. For the exposure “type of shift work”, the comparison was with those who always worked day shifts. For night shift characteristics (“frequency of night shifts”, “frequency of ≥3 consecutive night shifts” and “quick returns (<28 hours) from night shifts”) the comparisons were with employees who never worked night shifts (those who worked day and/or afternoon shifts but no night shifts). For “quick returns (<11 hours) from afternoon shifts” the analyses were restricted to those who never worked nights and the comparison was with those who never had quick returns. For “frequency of long (>45 hours) working weeks” the comparison was with those who never worked long working weeks (only working weeks of ≤40 hours). The estimated HR were always based on the exposure/shift work characteristics during the year preceding the outcome. We also performed trend tests, using the arithmetic average of the number of times (frequency) in each exposure category as a continuous variable in the regression model. The trend tests assessed the risk increases per ten additional times of the different aspects of night and shift work and long working weeks, such as ten additional times of night shifts.

We used SAS software, version 9.4 for Windows (SAS Institute Inc, Cary, NC, USA), with the statistical association at P<0.05 for all statistical analyses.

## Results

The baseline characteristics of the study participants at the inclusion year, according to the work schedules for the IHD cohort, are presented in table 1, and in supplementary material, www.sjweh.fi/article/4045, table S1 for the AF cohort. The baseline characteristics are based on the information from the inclusion year, but the work schedules are based on information from all the years the participants worked between 2008–2016. There were 6860 (22%) employees with permanent daytime work during the whole employment period, 11 181 (37%) employees with at least one afternoon shift (no night shifts), 11 240 (37%) employees with at least one night shift (excluding those working night only) and 1117 (4%) with permanent night shifts during the whole employment period. Compared with day workers, participants who were engaged in shift work with or without night shifts, or permanent night shifts, were more often men and from a country of birth outside the Nordic countries. Moreover, employees working shifts with or without night shifts were more often younger compared with day workers or permanent night workers. The proportion of nurses was highest among day workers and the proportion of nursing assistants was highest among permanent night workers (table 1).

**Table 1:** Baseline characteristics of the study participants (N=30 398) at the inclusion year in the cohort for ischemic heart diseases (ICD: I20-I25). Categorized by work schedule based on all the years the persons worked during 2008–2016.

Variables	Day work only ^a^ (N=6860)		Shift work, without night shifts ^b^ (N=11 181)		Shift work, with night shifts ^c^(N=11 240)		Night work only ^d^ (N=1117)	
			
	N	%	N	%	N	%	N	%
Sex								
Women	6431	94	9744	87	9624	86	825	74
Men	429	6	1437	13	1616	14	292	26
Age (years)								
≤40	1729	25	6206	55	7352	65	261	23
41–50	2099	31	2642	24	2450	22	348	31
>50	3032	44	2333	21	1438	13	508	46
Education								
Higher education (university ≥3 years)	3797	55	5635	50	7420	66	329	30
Upper secondary/Elementary or less	2926	43	5343	48	3623	32	735	66
Missing	137	2	203	2	197	2	53	4
Country of birth								
Sweden	5702	83	8595	77	8672	77	801	72
Nordic countries (except Sweden)	523	8	500	4	547	5	112	10
Europe (except Nordic countries)	127	2	343	3	400	4	45	4
Other countries	508	7	1743	16	1621	14	159	14
Profession								
Nurses	4740	69	5122	46	6967	62	366	33
Nursing assistants ^e^	2120	31	6059	54	4273	38	751	67

a Day work: starts after 06:00 and ends no later than 18:00.

b ≥1 afternoon shift (starts after 12:00 and ends later than 18:00, but not a night shift).

c ≥1 night shift (≥3 hours within 22:00 – 06:00 hours), but not only night work.

d Only night work (no day work or afternoon shifts).

e Assistant nurses, caregivers, accommodation assistants and personal assistants.

The 30 398 participants in the IHD cohort and the 30 475 participants in the AF cohort contributed to a total of 211 144 and 211 546 person-years, respectively. We observed a total of 223 incident IHD cases and 281 incident AF cases during up to eight years of follow-up 2009–2016. Of 223 cases of IHD, 175 were first identified from the national patient register and 48 cases from the regional VAL database. Of 281 cases of AF, 201 were first identified from the national patient register and 80 cases from the regional VAL database. The IHD cases included 76 cases of acute myocardial infarction, 124 cases of stable or unstable angina pectoris, and 23 cases of other IHD. The AF cases included 190 cases of paroxysmal or chronic AF and 91 cases of other AF and/or atrial flutter.

### Type and frequency of night work and risk of IHD and AF

Working permanent night shifts was associated with an increased risk of IHD the following year compared to those with permanent day work during the preceding year (HR 1.61, 95% CI 1.06–2.43) (table 2). The risk for IHD was higher among those who frequently worked night shifts (>120 times per year: HR 1.53, 95% CI 1.05–2.21), and who frequently worked ≥3 consecutive night shifts (15–20 times per year: HR 1.85, 95% CI 1.14–2.98), compared with those with no night work during the preceding year. The trend test indicated an increasing risk for IHD with increasing number of night shifts, expressed as risk per 10-night shifts (HR for beta 1.03, 95% CI 1.00–1.05) and with increasing 10 spells of ≥3 consecutive night shifts (HR for beta 1.06, 95% CI 1.00–1.20). That is, for every 10 additional night shifts and 10 additional spells of ≥3 consecutive night shifts, the risk for IHD increased by 3% and 6%, respectively (table 2). However, for spells of ≥3 consecutive night shifts, the increased risks were attenuated after additional adjustment for the total number of night shifts (table 2). All comparisons between type and frequency of night work and associations with AF demonstrated no increased risk in the adjusted model, also after additional adjustment for the total number of night shifts (table 3).

**Table 2:** Discrete-time proportional hazard models (adjusted) ^a^ for incident ischemic heart disease (ICD: I20-25, N=223) among healthcare employees during follow-up 2009–2016, contributing to a total of 211 144 person-years (PY). [HR=hazard ratio; CI=confidence interval.]

Exposure ^b^	Ischemic heart disease			

	PY	Cases (N)	Cases per 10 000 PY	HR (95% CI)
**Type of shift work**				
Always day shifts	72 461	88	12.1	Ref.
Day and afternoon shifts (no night shifts)	81 849	75	9.2	1.11 (0.79–1.55)
Day and/or afternoon shifts, and night shifts	41 503	25	6.0	0.93 (0.58–1.49)
Night shifts only	15 302	35	22.9	1.61 (1.06–2.43)
**Frequency of night shifts**				
Never night shifts ^c^	154 339	163	10.6	Ref.
1–19 (mean 8.2) times	14 437	5	3.5	0.70 (0.28–1.37)
20–62 (mean 37.2) times	14 243	5	3.5	0.66 (0.26–1.61)
63–120 (mean 94.9) times	11 390	15	13.2	1.11 (0.61–1.96)
>120 (mean 141.6) times	16 735	35	20.9	1.53 (1.05–2.21)
Trend test (risk increase per 10 times)				1.03 (1.00–1.05)
**Frequency of ≥3 consecutive night shifts**				
Never night shifts ^c^	155 408	163	10.5	Ref.
1–5 (mean 2.5) times	13 911	9	6.5	1.64 (0.63–2.44)
6–14 (mean 9.5) times	10 313	9	8.7	1.03 (0.50–2.10)
15–20 (mean 17.3) times	8988	20	22.3	1.85 (1.14–2.98)
>20 (mean 34.5) times	13 072	18	13.8	1.06 (0.64–1.74)
Trend test (risk increase per 10 times)				1.06 (1.00–1.20)
**Frequency of ≥3 consecutive night shifts.** Additionally adjusted for the total number of night shifts				
Never night shifts ^c^	155 408	163	10.5	Ref.
1–5 (mean 2.5) times	13 911	9	6.5	0.82 (0.35–1.89)
6–14 (mean 9.5) times	10 313	9	8.7	0.48 (0.16–1.43)
15–20 (mean 17.3) times	8988	20	22.3	0.63 (0.19–2.13)
>20 (mean 34.5) times	13 072	18	13.8	0.29 (0.07–1.21)
Trend test (risk increase per 10 times)				0.72 (0.53–1.00)
**Quick returns (<28 hours) from night shifts**				
Never night shifts ^c^	154 539	163	10.5	Ref.
1–12 (mean 5.4) times	14 915	6	4.0	0.83 (0.36–1.89)
13–37 (mean 22.8) times	13 008	7	5.4	0.93 (0.43–2.01)
38–71 (mean 58.4) times	14 547	23	15.8	1.33 (0.84–2.10)
>71 (mean 89.0) times	12 636	24	19.0	1.35 (0.87–2.10)
Trend test (risk increase per 10 times)				1.04 (1.00–1.08)
**Quick returns (<28 hours) from night shifts.** Additionally adjusted for the total number of night shifts				
Never night shifts ^c^	154 539	163	10.5	Ref.
1–12 (mean 5.4) times	14 915	6	4.0	0.73 (0.31–1.70)
13–37 (mean 22.8) times	13 008	7	5.4	0.58 (0.20–1.64)
38–71 (mean 58.4) times	14 547	23	15.8	0.47 (0.09–2.26)
>71 (mean 89.0) times	12 636	24	19.0	0.33 (0.04–2.63)
Trend test (risk increase per 10 times)				0.90 (0.72–1.13)
**Quick returns (<11 hours) from afternoon shifts.** Analyses were restricted to those who never worked night				
Never quick returns	69 360	75	10.8	Ref.
1–15 (mean 6.8) times	18 002	16	8.9	1.39 (0.78–2.47)
16–35 (mean 25.4) times	15 853	11	6.9	1.00 (0.50–1.96)
36–56 (mean 45.8) times	17 655	18	10.2	1.65 (0.97–2.81)
>56 (mean 70.2) times	21 116	25	11.8	1.68 (1.04–1.71)
Trend test (risk increase per 10 times)				1.04 (1.00–1.10)
**Frequency of long (>45 hours) working weeks**				
Never (only working weeks of ≤40 hours)	75 058	96	12.8	Ref.
1–3 (mean 1.8) times	30 883	26	8.4	1.11 (0.71–1.75)
4–6 (mean 4.9) times	19 414	7	3.6	0.42 (0.18–0.97)
7–11 (mean 8.9) times	26 026	13	5.0	0.63 (0.35–1.14)
>11 (mean 16.0) times	21 235	24	11.3	1.25 (0.78–1.99)
Trend test (risk increase per 10 times)				0.97 (0.72–1.31)

a Adjusted for sex, age (continuous), country of birth (Sweden; Nordic countries except Sweden; Europe except Nordic countries; other countries), education (higher education; upper secondary, elementary school or less) and profession (Nurses; Nursing assistants). Information on education was missing for 2% of the participants (no imputation was used to represent missing values).

b Based on the exposure during the year preceding the outcome. The cut-off points in categorizing exposure were based on 1–25^th^, 25–50^th^, 50–75^th^ and >75^th^ percentile in the distribution of the exposed group, except for type of shift work.

c Those who worked day and/or afternoon shifts, but no night shifts.

**Table 3:** Discrete-time proportional hazard models (adjusted ^a^) for incident atrial fibrillation (ICD: I48, N=281) among healthcare employees during follow-up 2009–2016, contributing to a total of 211 546 person-years (PY). [HR=hazard ratio; CI=confidence interval.]

Exposure ^b^	Atrial fibrillation			

	PY	Cases (N)	Cases per 10 000 PY	HR (95% CI)
**Type of shift work**				
Always day shifts	72 591	124	17.1	Ref.
Day and afternoon shifts (no night shifts)	81 924	101	12.3	1.21 (0.91–1.60)
Day and/or afternoon shifts, and night shifts	41 573	31	7.5	0.90 (0.59–1.36)
Night shifts only	15 430	25	16.2	0.78 (0.49–1.23)
**Frequency of night shifts**				
Never night shifts ^c^	154 543	225	14.6	Ref.
1–19 (mean 8.2) times	14 448	9	6.2	1.03 (0.52–2.02)
20–62 (mean 37.2) times	14 237	6	4.2	0.59 (0.26–1.34)
63–120 (mean 94.9) times	11 416	14	12.3	0.85 (0.49–1.46)
>120 (mean 141.6) times	16 902	27	16.0	0.72 (0.46–1.10)
Trend test (risk increase per 10 times)				0.98 (0.95–1.01)
**Frequency of ≥3 consecutive night shifts**				
Never night shifts ^c^	155 608	225	14.5	Ref.
1–5 (mean 2.5) times	13 920	9	6.5	0.92 (0.47–1.81)
6–14 (mean 9.5) times	10 339	8	7.7	0.74 (0.36–1.51)
15–20 (mean 17.3) times	9040	6	6.6	0.42 (0.18–0.95)
>20 (mean 34.5) times	13 179	21	15.9	1.02 (0.66–1.56)
Trend test (risk increase per 10 times)				0.97 (0.85–1.10)
**Frequency of ≥3 consecutive night shifts.** Additionally adjusted for the total number of night shifts				
Never night shifts ^c^	155 608	225	14.5	Ref.
1–5 (mean 2.5) times	13 920	9	6.5	1.20 (0.56–2.58)
6–14 (mean 9.5) times	10 339	8	7.7	1.27 (0.44–3.65)
15–20 (mean 17.3) times	9040	6	6.6	0.93 (0.21–3.99)
>20 (mean 34.5) times	13 179	21	15.9	2.66 (0.58–12.14)
Trend test (risk increase per 10 times)				1.43 (0.92–2.04)
**Quick returns (<28 hours) from night shifts**				
Never night shifts ^c^	154 741	225	14.5	Ref.
1–12 (mean 5.4) times	14 917	11	7.4	1.21 (0.65–2.25)
13–37 (mean 22.8) times	13 018	5	3.8	0.49 (0.20–1.21)
38–71 (mean 58.4) times	14 604	16	11.0	0.72 (0.43–1.20)
>71 (mean 89.0) times	12 765	23	18.0	0.77 (0.47–1.24)
Trend test (risk increase per 10 times)				0.99 (0.96–1.02)
**Quick returns (<28 hours) from night shifts.** Additionally adjusted for the total number of night shifts				
Never night shifts ^c^	154 741	225	14.5	Ref.
1–12 (mean 5.4) times	14 917	11	7.4	1.14 (0.59–2.20)
13–37 (mean 22.8) times	13 018	5	3.8	0.39 (0.11–1.35)
38–71 (mean 58.4) times	14 604	16	11.0	0.43 (0.06–2.95)
>71 (mean 89.0) times	12 765	23	18.0	0.39 (0.03–4.84)
Trend test (risk increase per 10 times)				0.96 (0.92–1.01)
**Quick returns (<11 hours) from afternoon shifts.** Analyses were restricted to those who never worked night				
Never quick returns	69 512	128	18.4	Ref.
1–15 (mean 6.8) times	18 025	24	13.3	1.47 (0.93–2.34)
16–35 (mean 25.4) times	15 831	10	6.3	0.71 (0.37–1.38)
36–56 (mean 45.8) times	17 654	17	9.6	1.08 (0.64–1.82)
>56 (mean 70.2) times	21 138	27	12.8	1.24 (0.79–1.93)
Trend test (risk increase per 10 times)				0.93 (0.72–1.20)
**Frequency of long (>45 hours) working weeks**				
Never (only working weeks of ≤40 hours)	75 266	134	17.8	Ref.
1–3 (mean 1.8) times	30 890	34	11.0	1.17 (0.79–1.73)
4–6 (mean 4.9) times	19 437	24	12.3	1.24 (0.78–1.97)
7–11 (mean 8.9) times	26 002	22	8.5	0.85 (0.53–1.35)
>11 (mean 16.0) times	21 313	16	7.5	0.57 (0.34–1.00)
Trend test (risk increase per 10 times)				0.69 (0.51–0.92)

a Adjusted for sex, age (continuous), country of birth (Sweden; Nordic countries except Sweden; Europe except Nordic countries; other countries), education (higher education; upper secondary, elementary school or less) and profession (Nurses; Nursing assistants). Information on education was missing for 2% of the participants (no imputation was used to represent missing values).

b Based on the exposure during the year preceding the outcome. The cut-off points in categorizing exposure were based on 1–25^th^, 25–50^th^, 50–75^th^ and >75^th^ percentile in the distribution of the exposed group, except for type of shift work.

c Those who worked day and/or afternoon shifts, but no night shifts.

^d^Analyses were restricted to those who never worked night.

### Quick returns and the risk of IHD and AF

Employees who frequently had quick returns (<28 hours) from night shifts had no significantly increased risk for IHD (HR 1.35; 95% CI 0.87–2.10) comparing >71 times per year to those with no night shifts. The trend test indicated that every ten additional quick returns from night shifts increased the risk for IHD by 4% (HR for beta 1.04, 95% CI 1.00–1.08). However, after additional adjustment for the total number of night shifts, the trend was attenuated (table 2). Analyses restricted to those who never worked night showed that employees with frequent quick returns (<11 hours) from afternoon shifts had an increased risk for IHD (HR 1.68; 95% CI 1.04–1.71) comparing >56 times per year to those with no quick returns. The trend test indicated an increasing risk for IHD with increasing ten times of quick returns from afternoon shifts (HR for beta 1.04, 95% CI 1.00–1.10). That is, for every ten additional quick returns from afternoon shifts, the risk for IHD increased by 4% (table 2). All comparisons between quick returns from night shifts or quick returns from afternoon shifts and associations with AF demonstrated no increased risk in the adjusted model, also after additional adjustment for the total number of night shifts (table 3).

### Long working weeks and the risk of IHD and AF

There was no statistically significant increased risk for IHD and AF among employees who often (>11 times per year) had long working weeks (>45 hours), compared to those with working weeks of ≤40 hours, and there was no trend of increasing risk estimates with the increasing number of times of long working weeks (table 2 and 3). However, the trend test indicated a decreasing risk for AF with increasing 10 times of long working weeks (HR for beta 0.69, 95% CI 0.51–0.92) (table 3).

## Discussion

In this prospective cohort study with registry-based exposure information, we observed an excess risk of incident IHD among healthcare employees who during the preceding year worked permanent night shifts, compared to those with permanent day work. There was also an excess risk of IHD among employees who worked night shifts >120 times during the preceding year compared to those with no night work, supported by a dose–response pattern. We also observed an excess risk of IHD related to frequent (15–20 times the preceding year) spells of ≥3 consecutive night shifts, but this effect was attenuated after additional adjustment for the total number of night shifts (to disentangle the effect of many nights from the effect of many consecutive nights). These findings suggest that the observed excess risk of IHD related to frequent spells of ≥3 consecutive night shifts, is more due to intensive night work per se and not specifically consecutive night work.

We acknowledge a certain degree of overlap between exposure groups, where permanent night workers are most likely overrepresented in the highest exposure groups. Therefore, the increased IHD risk for the highest exposure group of eg, “frequency of night shifts” may probably largely be attributed to intensive permanent night work. However, the group “night shifts only” also comprise employees with part-time work who are not necessarily part of the highest exposure groups.

There was no significant excess risk of IHD concerning frequent long working weeks of >45 hours. Among non-night workers we observed an excess risk of IHD related to frequent (>56 times the preceding year) quick returns (<11 hours) from afternoon shifts.

For “frequency of night shifts” the exposure group >120 times per year corresponded to a mean of 142 nights per year. In terms of night shifts per month, this would correspond to an average of >10 nights per month and a mean of 12 nights per month. For “quick returns from afternoon shifts” the exposure group >56 times per year corresponded to a mean of 70 times per year. This would similarly correspond to an average of >5 quick returns from afternoon shifts per month and a mean of 6 quick returns per month.

We did not observe any significant excess risk of AF related to any of these night and shift work patterns or long working hours. Instead, there was a trend of decreasing risk of AF with increasing frequency of long working weeks, which may reflect a healthy worker effect.

We are not aware of any earlier studies on the association between registry-based specific working hour characteristics and the risk of incident IHD but our results are in agreement with previous reviews and meta-analyses linking self-reported working hour data to the risk of IHD (13, 15, 17). For example, the meta-analysis by Cheng et al (17) suggested an association between the risk of IHD and increasing duration of shift work, supported by a positive dose–response pattern. Vyas et al (13) in a pooled subgroup analysis, reported the highest increase in IHD risk (41%) for employees with permanent night shifts. In the present study, we also found the highest risk of IHD (61%) associated with permanent night shift work and our results indicate that the intensity of night work, in general, is of most importance for the IHD risk. In a recent evaluation (review of reviews) by Rivera et al (28) the quality of the epidemiologic evidence between shift work (defined as any work outside the standard daytime work hours) and health conditions, was estimated as low for IHD and myocardial infarction and very low for events of CVD overall. However, their evaluation did not differentiate between evidence for different types of shift work, various intensity of shift work or for night work specifically. In the present study the association with IHD risk was strongest for intensive night work.

Some possible biological mechanisms may explain the association between night and shift work and IHD risk. The classic opinion proposes that circadian disruption caused by shift work results in the desynchronization of endogenous and exogenous components and disturbances of the cardiovascular system (29). It has also been proposed that shift work is associated with increased psychosocial stress (6, 9), leading to hypersecretion of cortisol and catecholamines, which may contribute to disorders increasing the risk for IHD. Furthermore, smoking, physical inactivity in leisure time, changes in appetite-regulating hormones and other factors leading to an unhealthy diet and being obese, increased alcohol consumption, the likelihood of ignoring symptoms of disease, elevated blood lipids, and high blood pressure are some underlying factors that have been suggested to be involved in the association between shift work and IHD (9–11). Sleep deprivation and insufficient recovery, related to consecutive night shifts and quick returns, could be another possible pathway from night shift work to the increased risk of IHD. Several epidemiological and experimental studies including female hospital employees found that the number of consecutive night shifts was associated with progressive changes in hormones involved in circadian rhythms, such as melatonin and cortisol, and heart rate variability (30–32), and thereby might increase the risk of IHD. In the previous studies, working ≥3 consecutive night shifts was associated with a decrease in melatonin among hospital employees in Canada (30) and Denmark (32). Furthermore, a prospective, longitudinal study of hospital nurses suggests that nurses must be allowed more than two days off work on changing from night shifts to other shifts to adjust their circadian rhythms of salivary cortisol levels (33). Our findings suggest that the intensity of night shifts, in general, seems to be a more decisive factor behind the increased risk of quick returns from nights shifts than the number of consecutive shifts. However, in our previous study based on the same cohort as in the present study, the risk of incident cerebrovascular disease was more influenced by consecutive night shift work per se (25). In the present study, we also found an association between a short recovery time (<11 hours) after the afternoon shifts and IHD among non- night workers. A short recovery time between the shifts is associated with increased sleepiness and a higher level of perceived stress (13), insulin resistance (6), and activation of the immune system with the result of inflammation (34), which in turn may contribute to the development of IHD. Furthermore, a prospective study suggests that incomplete recovery from work is an aspect of the overall risk profile of CVD mortality among industrial employees (35).

Our study did not observe any association between frequent long working weeks (>45 hours) and the risk of IHD. However, our study does not rule out an elevation of IHD risk associated with frequent very long working weeks. A recent meta-analysis observed an association between working on average ≥55 hours per week and IHD (16). Due to the small sample size in the category of frequent long working weeks of ≥55 hours, our study lacked the power to analyze it.

Our results do not support the findings of the previous study by Wang et al (19) that employees with permanent night shifts and working an average of 3–8 nights per month increase the risk of incident AF. In their study, the exposure information was self-reported and assessed at the baseline only which could explain some of the differences in the results. It is intriguing though that we could observe an effect of night shift work on IHD but not AF. These outcomes share several similar risk factors, but our results indicate that the risk of AF is not affected in the same way by intensive night shift work.

In this study, we chose a time window of one year for the exposure, to compromise between acute and chronic effects on IHD and AF. However, this time window does not exclude those employees who may have belonged to other exposure groups previously, since the exposure classification was updated annually to account for variations over time. The one-year time window reflects the exposure from a combination of acute working time triggers the previous year and cumulative demanding working times, since the employees may have worked in a similar way in previous years.

### Strengths and limitations

The main strength of this study is very detailed registry-based data on working hours during the employment period. The data was not from the employees themselves, which avoids the risk that people forget details about their working hours back in time. Thus, our study was not biased by self-reported and/or retrospective estimations. Another strength is that data on health outcomes are retrieved from registers of good quality and that the cohort consists of employees in the healthcare sector where the percentage of shift and night workers is high. Moreover, healthcare employees represent a homogeneous group with less variability in potential confounders related to socioeconomic position. We had a coverage of cases from both in- and outpatient hospital care services and also from outpatient visits at the regional general practice in Stockholm.

Some limitations include a lack of information on important lifestyles and other individual risk factors associated with IHD and AF, such as smoking habits, physical inactivity, hypertension, and diabetes, that can act as confounders or mediators. We also lacked information on physical and mental workload, such as the possibility of sleep during on-call shifts, and on chronotype and personal work hour preferences of the participants. Our study includes only healthcare employees, with the majority having constantly changing schedules over the employment period. Therefore, our results may not apply to populations with other occupations. Additionally, we did not account for the possible healthy worker effect, which would underestimate the actual risk. Employees with health problems may need to reduce their work intensity over time or be transferred from night to day work.

### Concluding remarks

In this prospective cohort study of >30 300 healthcare employees with registry-based data on working hours, we found that intensive night work, especially working permanent night shifts and frequent night shifts is associated with an increased risk of incident IHD but not AF. Among non-night workers, we observed an excess risk of IHD related to frequent quick returns from afternoon shifts.

These findings add to previous observations from this cohort which suggest that there is an increased risk of preterm birth and cerebrovascular disease in relation to intensive night work, especially working many spells of consecutive night shifts and short recovery after night shifts. When healthcare employees are engaged in night work, their health might benefit from avoidance of permanent night shift work, a restriction of the total number of night shifts per year, and an adequate recovery time after any shift. Ideally, future studies on night work in relation to IHD and AF should include other occupational groups and combine objectively measured working hours and personal characteristics like chronotype and working hour preferences, as well as working conditions (eg, workload).

## Supplementary material

Supplementary material
