# Supplementary material for: Night and shift work characteristics and incident ischemic heart disease and atrial fibrillation among healthcare employees – a prospective cohort study
Source: Scand J Work Environ Health. 2022 Oct 1;48(7):520–9. doi: 10.5271/sjweh.4045 (PMC10539110; doi:10.5271/sjweh.4045)
Supplement: Supplementary material [file SJWEH-48-520-S001.pdf]

Night and shift work characteristics and incident ischemic heart disease and atrial fibrillation among healthcare employees – a prospective cohort study[1]

by Manzur Kader, PhD, Jenny Selander, PhD, Tomas Andersson, BSc, Maria Albin, MD, Theo Bodin, MD, Mikko Härmä, MD, Petter Ljungman, MD, Carolina Bigert, MD, [2]

1. Supplementary material
2. Correspondence to: Carolina Bigert, Institute of Environmental Medicine, Karolinska Institutet, Solnavägen 4 10th floor, SE-113 65 Stockholm, Sweden. [E-mail: carolina.bigert@ki.se].

Supplementary Table S1: Baseline characteristics of the study participants (N=30 475) at the inclusion year in the cohort for atrial fibrillation (ICD: I48).
Categorized by work schedule based on all the years the person worked during 2008–2016.

	Day work only[a] (n= 6893)		Shift work, without night shifts[b] n= 11 197		Shift work, with night shifts[c] n= 11 254		Night work only[d] (n= 1131)	
	N	%	N	%	N	%	N	%
Sex								
Women	6454	94	9757	87	9635	86	834	74
Men	439	6	1440	13	1619	14	297	26
Age								
≤40 years	1727	25	6193	55	7344	65	260	23
41-50 years	2093	30	2644	24	2453	22	351	31
>50 years	3073	45	2360	21	1457	13	520	46
Education								
Higher education (university ≥3 years)	3810	55	5641	50	7423	66	333	30
Upper secondary / Elementary or less	2946	43	5351	48	3633	32	746	66
Missing	*137*	*2*	*205*	*2*	*198*	*2*	*52*	*4*
Country of birth								
Sweden	5721	83	8601	77	8686	77	809	72
Nordic countries (except Sweden)	531	8	506	4	549	5	112	10
Europe (except Nordic countries)	128	2	344	3	401	4	47	4
Other countries	513	7	1746	16	1618	14	163	14
Profession								
Nurses	4761	69	5128	46	6969	62	370	33
Nursing assistants[e]	2132	31	6069	54	4285	38	761	67

[a]Day work: starts after 06:00 and ends no later than 18:00
[b]At least one afternoon shift (starts after 12:00 and ends later than 18:00, but not a night shift)
[c]At least one night shift (at least three hours within 22:00 – 06:00 hours), but not only night work
[d]Only night work (no day work or afternoon shifts)
[e]Assistant nurses, caregivers, accommodation assistants and personal assistants
